# ESI-MS/MS Identification of a Bradykinin-Potentiating Peptide from Amazon *Bothrops atrox* Snake Venom Using a Hybrid Qq-*oa*TOF Mass Spectrometer

**DOI:** 10.3390/toxins5020327

**Published:** 2013-02-18

**Authors:** Antonio Coutinho-Neto, Cleópatra A. S. Caldeira, Gustavo H. M. F. Souza, Kayena D. Zaqueo, Anderson M. Kayano, Rodrigo S. Silva, Juliana P. Zuliani, Andreimar M. Soares, Rodrigo G. Stábeli, Leonardo A. Calderon

**Affiliations:** 1 Center of Biomolecules Study Applied to Health, Fiocruz Rondônia, Oswaldo Cruz Foundation, Porto Velho, RO 76820-245, Brazil; E-Mails: antonio.dnabrasil@gmail.com (A.C.-N.); cleobiol@gmail.com (C.A.S.C.); kayena@gmail.com (K.D.Z.); kayano@unir.br (A.M.K.); simoesdg@gmail.com (R.S.S.); zuliani.juliana@gmail.com (J.P.Z.); andreimarsoares@gmail.com (A.M.S.); stabeli@fiocruz.br (R.G.S.); 2 MS Applications Development Laboratory, Waters Corporation, Alphaville, SP 06455-020, Brazil; E-Mail: gustavo_souza@waters.com; 3 Medicine Department, Federal University of Rondônia, Porto Velho, RO 76801-059, Brazil

**Keywords:** bioactive peptide, BPP, pyroglutamic acid, pyrrolidonecarboxylic acid, *de novo* peptide sequencing

## Abstract

A bradykinin-potentiating peptide (BPP) from Amazon *Bothrops atrox* venom with *m/z* 1384.7386 was identified and characterized by collision induced dissociation (CID) using an ESI-MS/MS spectra obtained in positive ion mode on a hybrid Qq-*oa*TOF mass spectrometer, Xevo G2 QTof MS (Waters, Manchester, UK). *De novo* peptide sequence analysis of the CID fragmentation spectra showed the amino acid sequence ZKWPRPGPEIPP, with a pyroglutamic acid and theoretical monoisotopic *m*/*z* 1384.7378, which is similar to experimental data, showing a mass accuracy of 0.6 ppm. The peptide is homologous to other BPP from *Bothrops moojeni* and was named as BPP-BAX12.

## 1. Introduction

Snake venoms have been recognized as an extensible source of bioactive peptides with potential biotechnological applications in medicine [1]. Due to their high degree of target specificity, venom toxins have been increasingly used as lead compounds in the development of drug prototypes [2]. One of the most successful examples has been Captopril^®^, an antihypertensive drug based on a bradykinin-potentiating peptide (BPP) isolated from Brazilian *Bothropoides* (*Bothrops*) *jararaca* venom [3,4]. The BPP family comprises a class of angiotensin-I converting enzyme (ACE) inhibitors with different lengths (5 to 14 amino acid residues) found in venoms produced by snakes, scorpions, spiders and amphibians [5]. Generally, BPPs have a conserved *N*-terminal pyroglutamate residue (Z) and two consecutive proline residues at the *C*-terminal region [6,7]. This work describes the identification and characterization of a new BPP from Amazon *Bothrops atrox* snake venom. 

## 2. Materials and Methods

### 2.1. Venom

*Bothrops atrox* specimens collected around the city of Porto Velho, State of Rondônia, Brazil were kept at Fiocruz Rondônia bioterium in order to be used for venom production under authorization emitted by IBAMA (licence number 27131-1) and CGEN (licence number 010627/2011-1). The crude venom was dehydrated and stored at a temperature of −20 °C in the Amazon Venom Bank at CEBio. 

### 2.2. Peptide Isolation

The purification of BPP-BAX12 was performed using 50 mg of crude venom, which was divided fractioned into two fractions on a size exclusion chromatography column using a Superdex peptide-10/300GL column (GE Healthcare) equilibrated with 50 mmol/L Tris-HCl buffer (pH 7.4) and carried out at a flow rate of 0.5 mL/min. The second fraction produced, which was related to peptides, was re-chromatographed under the same conditions resulting in eight fractions. The fourth fraction (37–43 min) was then lyophilized and stored for MS/MS analysis.

### 2.3. MS Parameters and Data Acquisition

ESI-MS spectra were obtained in positive ion mode on a hybrid Qq-*oa*TOF mass spectrometer—Xevo G2 QTof MS (Waters, Manchester, UK). Typical ESI-MS conditions were done in positive mode as follow: source temperature 80 °C, capillary voltage 2.8 kV, and cone voltage 35 V, resolution mode with an analogic-to-digital converter (ADC) mode, detector at 2825 V previously adjusted with leukine enkephalin (Leu-Enk) solution at 2 ng/μL. The instrument was automatically calibrated with sodium iodide solution through IntelliStart, integral part of MassLynx 4.1v acquisition software (Waters, Manchester, UK). Samples were re-suspended in a vial with a solution containing equal parts of water and methanol with 0.1% of formic acid for each sample to proceed ESI(+)-MS analysis. These solutions were then injected at a flow rate of 500 nL/min, using the fluid system installed in the Xevo G2 QTof MS panel controlled by the IntelliStart software and MS tune page. All MS spectra were acquired over the *m/z* 50–2000. MS/MS acquisition was performed using the quadrupole with high discrimination for each *m/z* of interest. The collision energy was applied to the selected precursor ion and a collision-induced dissociation (CID) at the T-Wave collision cell filled with argon gas was used. 25 eV was applied to the collision cell depending on the precursor ion dissociation characteristics. 

### 2.4. MS/MS Analysis

The MS/MS spectra were de-convoluted using MaxEnt 3 software (Waters, Manchester, UK) and then transferred to a PepSeq application into BioLynx software package and a Microsoft Excel file with data up to 120 counts in order to proceed with manual evaluation. The identification of the most common diagnostic peptide fragment ions (a^+^, b^+^, y^+^-type) currently observed in low energy collisions and immonium ions for *de novo* peptide sequencing were performed manually using the program Microsoft Excel with data of monoisotopic mass of common and less common amino acid residues, terminal groups and post-translational modifications for the use in mass spectrometry calculated using the following atomic masses of the most abundant isotope of the elements: C = 12.0000000, H = 1.0078250, N = 14.0030740, O = 15.9949146, F = 18.9984033, P = 30.9737634, S = 31.9720718, Cl = 34.9688527, Br = 78.9183361. Fragments with intensity higher than 200 counts and mass accuracy between 0 and ± 17 ppm, according to the equation 1, was used for *de novo* peptide sequencing.

Mass accuracy (ppm) = 1,000,000 × (theoretical mass − measured mass)/theoretical mass

MassSeq application and *de novo* sequencing analysis and interpretation tool of the BioLynx software package was used in order to confirm manual analysis using the following peptide sequencing parameters: *m*/*z* tolerance of 0.03 for peptide and fragments and intensity threshold of 0.003%.

## 3. Results and Discussion

**Figure 1:**
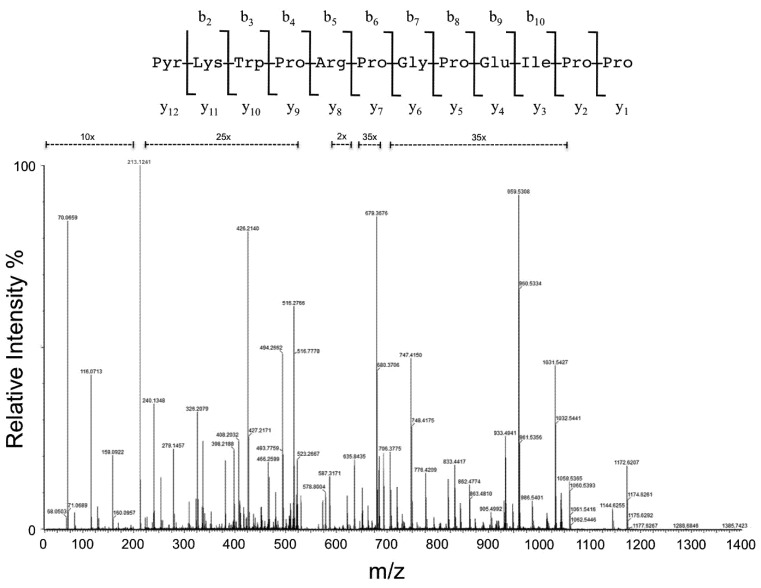
Collision-induced dissociation spectra of BPP-BAX12. The deduced sequence is shown at the top of the MS/MS profile. The inset shows the assigned peptide sequence.

The mass spectrometric analysis of the fourth chromatographic fraction reveals a high intensity doubly protonated ion peak at *m/z* 692.8732 [M + 2H]^2+^. The ion was selected and submitted to collision-induced dissociation (CID) with argon gas resulting in a mass spectrum (Figure 1), which was submitted to the identification of a^+^, b^+^, and y^+^-type diagnostic fragments and immonium ions for *de novo* peptide sequence (Table 1, Table 2) [8]. The analysis revealed a 12 residue proline-rich peptide (Pyr-Lys-Trp-Pro-Arg-Pro-Gly-Pro-Glu-Ile/Leu-Pro-Pro) with a conserved consecutive two proline residues at the *C*-terminal region, a characteristic of the BPP family of ACE inhibitors [6,7], and a *N*-terminal pyroglutamic acid (Pyr), which could be derived from glutamine or glutamic acid residues, as observed in other currently described snake venom BPPs from Bothrops species. The measured peptide monoisotopic mass (1384.7386) and theoretical (1384.7378) was very similar, showing a mass accuracy of 0.6 ppm, which was also observed for the identified diagnostic fragment ions (Table 1, Table 2), thus showing the high precision of the analysis. Sequence similarity showed that the peptide is homologous to other BPP described for *B. moojeni* venom [6] and similar to others from *Bothrops neuwiedi* [1,9], *B. leucurus*, *B. erythromelas*, *B. alternatus* [10], *B. insularis* [1,10,11], *B. jararaca* [12,13], *B. jararacussu* [1,10,14], *B. cotiara* [13], and *B. fonsecai* [13] (Table 3). This peptide was named as Bradykinin-potentiating peptide BAX12.

**Table 1:** Diagnostic peptide fragments (b, a and y-type ions) obtained by collision-induced dissociation with argon gas used for *de novo* peptide sequencing of BPP-BAX12.

Aminoacid Residue	Fragment	Theoretical (*m*/*z*)	Measured (*m*/*z*)	Intensity (counts)	Accuracy (ppm)	Fragment	Theoretical (*m*/*z*)	Measured (*m*/*z*)	Intensity (counts)	Accuracy (ppm)	Fragment	Theoretical (*m*/*z*)	Measured (*m*/*z*)	Intensity (counts)	Accuracy (ppm)
Z	b_1_	112.0399	-	-	-	a_1_	84.0688	-	-	-	y_12_	1384.7377	1384.7548	1.6 × 10^2^	11.7
K	b_2 _*	223.1083	223.1082	2.7 × 10^3^	0.2	a_2_ *	195.1134	195.1134	4.0 × 10^3^	−0.2	y_11_	1273.7057	1273.6853	1.6 × 10^2^	16.0
	b_2_	240.1348	240.1348	5.2 × 10^4^	0.1	a_2_	212.1399	212.1405	6.1 × 10^3^	−2.9	y_11 _*	1256.6792	-	-	-
W	b_3_	426.2141	426.2140	1.2 × 10^5^	0.3	a_3_	398.2192	398.2188	3.3 × 10^4^	1.0	y_10_	1145.6107	1145.6276	2.0 × 10^5^	14.8
P	b_4_	523.2669	523.2667	2.9 × 10^4^	0.4	a_4_	495.2720	495.2684	3.1 × 10^4^	7.2	y_9_	959.5314	959.5308	9.9 × 10^4^	0.6
R	b_5 _*	662.3415	622.3417	7.0 × 10^3^	−0.4	a_5 _*	634.3466	634.3317	3.7 × 10^4^	23.4	y_8_	862.4786	862.4774	1.3 × 10^4^	1.4
	b_5_	679.3680	679.3676	9.3 × 10^4^	0.6	a_5_	651.3731	651.3705	1.2 × 10^4^	4.0	y_8 _*	845.4520	845.4680	6.0 × 10^3^	−18.9
P	b_6_	776.4208	776.4209	1.7 × 10^4^	−0.2	a_6_	748.4259	748.4175	3.0 × 10^4^	11.2	y_7_	706.3775	706.3775	2.3 × 10^4^	0.0
G	b_7_	833.4422	833.4417	1.9 × 10^4^	−0.6	a_7_	805.4473	805.4473	1.8 × 10^3^	0.0	y_6_	609.3248	609.3238	3.6 × 10^2^	1.6
P	b_8_	930.4950	930.4941	8.4 × 10^3^	1.0	a_8_	902.5001	902.5016	1.2 × 10^3^	−1.7	y_5_	552.3033	552.3032	4.8 × 10^3^	0.2
E	b_9 _#	1041.5273	1041.5265	2.0 × 10^3^	0.8	a_9 _#	1013.5321	1013.5197	4.4 × 10^2^	12.2	y_4_	455.2505	455.2492	3.7 × 10^2^	2.8
	b_9_	1059.5376	1059.5365	5.0 × 10^5^	1.0	a_9_	1031.5427	1031.5427	4.9 × 10^4^	0.0	y_4 _#	437.2399	437.2360	6.3 × 10^3^	9.0
I/L	b_10_	1172.6206	1172.6207	6.6 × 10^5^	−0.1	a_10_	1144.6257	1144.6255	2.1 × 10^5^	0.2	y_3_	326.2081	326.2079	4.9 × 10^4^	0.7
P	b_11_	1269.6734	-	-	-	a_11_	1241.6785	-	-	-	y_2_	213.1241	213.1241	3.8 × 10^6^	−0.1
P	b_12_	1366.7261	-	-	-	a_12_	1338.7312	-	-	-	y_1_	116.0712	116.0713	1.6 × 10^5^	−1.2

Immonium ions detected Theoretical *m*/*z*, measured *m*/*z* (accuracy in ppm): Z 84.04496, 84.0446 [4.3]; K(–NH_3_) 84.08129, 84.08144 [−1.3]; R(–NH_3_) 112.08746, 112.0877 [−2.2]; W 159.09220, 159.0922 [0.0]; P 70.06568, 70.0659 [3.1]; I/L 86.09698, 86.0970 [−0.2]. * Loss of a neutral ammonia (NH_3_) molecule from K or R side chains. **# ** loss of a neutral H_2_O molecule from E side chain.

**Table 2:** Diagnostic internal fragments (b and a-type ions) ions obtained by collision-induced dissociation with argon gas used for *de novo* peptide sequencing of BPP-BAX12.

Fragments	b-Type ions				a-Type ions			
	Theoretical (*m*/*z*)	Measured (*m*/*z*)	Intensity (counts)	Accuracy (ppm)	Theoretical (*m*/*z*)	Measured (*m*/*z*)	Intensity (counts)	Accuracy (ppm)
KWPRPGP	819.4629	-	-	-	791.46799	-	-	-
KWPRPGP *	802.4364	802.4401	2.3 × 10^2^	−4.6	774.44149	-	-	-
KWPRPG	722.4102	-	-	-	694.41529	-	-	-
KWPRPG *	705.3836	705.3939	8.2 × 10^2^	−14.6	677.38869	677.3793	1.0 × 10^3^	13.9
KWPRP	665.3887	-	-	-	637.39379	-	-	-
KWPRP *	648.3622	-	-	-	620.36729	-	-	-
KWPR	568.3359	-	-	-	540.34099	540.3436	1.7 × 10^2^	−4.8
KWPR *	551.3094	-	-	-	523.31449	-	-	-
KWP	412.2349	412.2426	4.9 × 10^2^	−18.7	384.23999	-	-	-
KWP *	395.2083	-	-	-	367.21339	-	-	-
KW	315.1821	315.1797	2.2 × 10^2^	7.6	287.18719	-	-	-
KW *	298.1556	298.1567	6.3 × 10^2^	−3.7	270.16069	270.1608	1.9 × 10^3^	−0.4
WPRPGPEIP	1030.5474	1030.5469	1.0 × 10^3^	0.5	1002.5525	1002.5590	1.3 × 10^2^	−6.5
WPRPGPEIP *	1013.5209	1013.5197	4.4 × 10^2^	1.1	985.5259	-	-	-
WPRPGPEI	933.4946	933.4941	2.8 × 10^4^	0.5	905.4997	905.4992	5.0 × 10^3^	0.5
WPRPGPEI *	916.4681	916.4681	2.5 × 10^3^	0.0	888.4731	888.4796	9.8 × 10^2^	−7.3
WPRPGPE	820.4106	820.4102	1.5 × 10^4^	0.5	792.4157	792.4160	3.4 × 10^3^	−0.4
WPRPGPE *	803.3841	803.3862	1.5 × 10^3^	−2.7	775.3891	775.3857	6.3 × 10^2^	4.4
WPRPGP	691.3680	691.3715	7.7 × 10^2^	−5.1	663.3731	-	-	-
WPRPGP *	674.3415	-	-	-	646.3465	646.3524	3.0 × 10^2^	−9.1
WPRPG	594.3152	594.3206	2.4 × 10^3^	−9.1	566.3203	-	-	-
WPRPG *	577.2887	577.2831	1.9 × 10^2^	9.6	549.2937	-	-	-
WPRP	537.2938	-	-	-	509.2989	-	-	-
WPRP *	520.2673	-	-	-	492.2723	492.2757	1.6 × 10^2^	−6.8
WPR	440.2410	440.2399	4.6 × 10^3^	2.5	412.2461	-	-	-
WPR *	423.2145	423.2138	2.2 × 10^3^	1.5	395.2195	395.2174	8.6 × 10^2^	5.4
WP	284.1399	-	-	-	256.1450	256.1468	5.5 × 10^2^	−7.1
PRPGPEIP	844.4681	844.4672	7.8 × 10^3^	1.1	816.4732	-	-	-
PRPGPEIP *	827.4416	827.4381	1.6 × 10^2^	4.2	799.4466	-	-	-
PRPGPEI/RPGPEIP	747.4153	747.4150	5.1 × 10^4^	0.4	719.4204	719.4196	1.3 × 10^4^	1.1
PRPGPEI */RPGPEIP *	730.3888	730.3976	4.4 × 10^3^	−12.1	702.3938	702.4028	1.4 × 10^3^	−12.7
PRPGPE	634.3313	634.3317	3.7 × 10^4^	−0.6	606.3364	606.3362	1.1 × 10^4^	0.3
PRPGPE *	617.3048	617.3059	3.4 × 10^3^	−1.9	589.3098	589.3193	2.2 × 10^3^	−16.0
PRPGP	505.2887	505.2886	1.6 × 10^3^	0.2	477.2938	477.295	3.9 × 10^2^	−2.5
PRPGP *	488.2622	488.2668	1.4 × 10^2^	−9.5	460.2672	-	-	-
PRPG/RPGP	408.2359	408.2354	2.2 × 10^3^	1.2	380.2410	380.2346	5.6 × 10^2^	16.8
PRPG */RPGP *	391.2094	-	-	-	363.2144	-	-	-
PRP	351.2145	351.2112	2.1 × 10^3^	9.4	323.2196	-	-	-
PRP *	334.1880	334.1869	6.7 × 10^2^	3.2	306.1930	-	-	-
PR/RP	254.1617	254.1616	2.2 × 10^4^	0.4	226.1668	-	-	-
PR/RP *	237.1352	237.136	2.8 × 10^3^	−3.6	209.1402	209.1388	4.1 × 10^2^	6.9
RPGPEI	650.3626	650.3616	4.7 × 10^3^	1.5	622.3677	-	-	-
RPGPEI *	633.3361	-	-	-	605.3411	605.3397	1.2 × 10^3^	2.4
RPGPE	537.2785	537.2814	2.9 × 10^3^	−5.4	509.2836	509.2862	2.2 × 10^3^	−5.1
RPGPE *	520.2520	520.2515	9.3 × 10^2^	0.9	492.2570	492.2552	6.2 × 10^2^	3.7
RPG	311.1832	-	-	-	283.1883	-	-	-
RPG *	294.1567	294.1559	2.4 × 10^2^	2.6	266.1617	-	-	-
IP	211.1446	211.1445	3.1 × 10^3^	0.5	183.14969	183.1503	1.2 × 10^3^	−3.3

* Loss of a neutral ammonia (NH_3_) molecule from K or R side chains.

**Table 3:** Sequence alignment between BPP-BAX12 and Pyroglutamate peptides/Bradykinin-potentiating (BPPs) sequences from others Bothrops species.

BPP name	Sequence	Bothrops specie	Reference
BPP-BAX12	ZKWPRPGPEIPP	*Bothrops atrox*	this work
-	ZKWPRPGPEIPP	*B. moojeni*	[6]
-	ZNWPRPGPEIPP	*B. moojeni*	[6]
BPP3_BOTNU, BPP13_BOTMO, BPP13_BOTLC, BPP13_BOTER, BPP13_BOTAL, BNP_BOTIN, BNP2_BOTJA, BNP1_BOTJA, Q8QG90_BOTIN, BNP_BOTJR **^#^**	ZGGWPRPGPEIPP	*B. neuwiedi*, *B. moojeni*, *B. leucurus*, *B. erythromelas*, *B. alternatus*, *B. insularis*, *B. jararaca*, *B. jararaca*, *B. insularis*, *B. jararacussu*	[1,9,10,11,12,13]
BPP-13a	ZGGWPRPGPEIPP	*B. cotiara*, *B. fonsecai*	[14]
BPP-13b	ZGGLPRPGPEIPP	*B. cotiara*, *B. fonsecai*	[14]

**^#^** Entry name from UniProtKB.

## 4. Conclusions

Recent papers on venomics [15], proteome [16] and transcriptome [17] of *B. atrox* snake have shown an absence of BPP structures. However, only a single cluster that matched a 5' untranslated region of a BPP mRNA from *B. jararacussu* snake was found [11]. The BAX12 is the first peptide belonging to the BPP family of ACE inhibitor described for *Bothrops atrox*. The complete homology between BPP-BAX12 from *Bothrops moojeni* [6] and others BPPs could provide interesting information regarding the evolutionary relationship between Bothrops snake species.
